# Exploring the Concept of ‘Positive Ageing’ in the UK Workplace—A Literature Review

**DOI:** 10.3390/geriatrics3040072

**Published:** 2018-10-18

**Authors:** Diane Keeble-Ramsay

**Affiliations:** Positive Ageing Research Institute and Lord Ashcroft International Business School, Anglia Ruskin University, Rivermead Campus, Chelmsford CM1 1SQ, UK; diane.keeble-allen@anglia.ac.uk; Tel.: +44-845-196-6844

**Keywords:** positive ageing, older workers, demographic change

## Abstract

The participation rate of older people in the labour market is forecast to increase due to demographic changes afoot. For example, low fertility rates, higher life expectancy, and increases in the retirement age will affect labour availability. The working-age population trends indicate that the age group 55–64 years will expand. This trend is bolstered by policy debate about the sustainability of economic and social support systems for the wider population and necessary strategies to keep older workers in labour markets. Within the UK, as the statutory pension age is placed now at 67, changes affecting the national default retirement age (previously age 60 for women and 65 for men) already mean that many older workers will feature in workplaces past historical expectations. A lack of sensitivity about the adjustments older workers needed, due to age-related changes in health and functional capacities, attests the demoted valuing of ageing workers. Despite a rise in the importance of wisdom across cultures, the significance of experience that comes with ageing, however, has become less revered within the UK resulting in less than the institutional promotion of Positive Ageing might depict. This paper draws from a structured review of literature (SLR) which seeks to address the question of what is currently identified as ‘Positive Ageing’ to consider what contributions can be found in current literature that may represent these changes in the UK. The paper concludes that demographic change has stimulated significant re-thinking of workplace strategies for the maintenance of health and well-being of ageing workers at national or governmental policy levels. To ensure sustainability, workability, and productivity in work, however, the concept of Positive Ageing towards later life might be furthered despite that, at the organizational level, its enactment remains incomplete currently post retirement age.

## 1. Introduction

Resulting from population ageing predictions for the coming decades, it has become apparent that there will be future demographic upheaval both in the wider society and as a result within the labour market. To exemplify, for the first time, by mid-2014 in the United Kingdom, the average age exceeded 40 [1]. By the year 2040, for both genders, it has been predicted that nearly one in seven will be over the age of 75. One in three babies born can expect to live to 100 [2]. This requires society to adapt. Responses to such change by delaying the age of retirement, however, potentially significantly impacts upon the composition of the workforce. To which the needs and values of older workers in terms of their health, the need for meaningful work, autonomy and desire to seek further training represents an extra dynamic. One that will influence the very nature of the workplace that might have been otherwise overlooked by UK employers [3].

It is apparent, however, to date, that UK employers are not making the most of any opportunities that might be afforded by the potential of an ageing population. Too many within the UK are forced out of work by factors, such as management cultures that are not conducive to ageing, alongside poor health [3]. Furthermore, there has been maladaptation within the changing labour market towards caring responsibilities of and for older workers. An apparent abandonment of UK aspirations towards ‘best practice’ people management strategies in the 21st century and limited trades unions’ power may have compounded the inability and/or desirability of people to continue working in their autumn years. Unhealthy lifestyle behaviours exist in the UK workplace, which may be formed both within workplace practices, for example through inactive, sedentary working. Also, further from work intensification, where stress levels rise, and mental health diminishes. The health implications associated with inactivity and sedentary lifestyles, fashioned from workplace practices that raise stress levels and poor mental health, for example depression or anxiety, which may predominate working lives, raise concerns [4]. There is further anxiety that there are those who have been unable to participate in the UK workplace, based upon there being discriminatory employment practices for the over 50s. Potentially they fall outside historical economic measures, resulting from restructuring or corporate downsizing, which perceives their pension needs or attracted salary as ‘expensive employees’. This then leads towards this age cohort becoming (considered as) economically inactive [5,6]. There is considerable fear about mental health problems evolving from the current workplace (burnout/exhaustion) which then transfers into this period of economic inactivity or retirement, all of which is detrimental. However, workplace ageism prevails within the UK currently [7]. It is ubiquitous.

To remedy some of this, the concept of ‘Positive Ageing’ has been coined. This term might be viewed as a way of living that marries the mental state with physical wellbeing. By representing constructive attitudes towards ageing, since Positive Ageing incorporates the physical, psychological and social well-being needs of people as they age within the workplace, the concept of ‘Positive Ageing’ adopted within this study seeks to facilitate further thinking about later life challenges. Being physically active has been already accepted within ‘Active Ageing’ as contributory towards a positive mental wellbeing and the limitation of some of the potential detriments historically associated with ageing [4]. Given the above stated problems associated with inactive, sedentary working, the links between activity and mental wellbeing when reviewing work-based matters, this concept of ‘Active Ageing’ might be viewed further as an integral, or contributory, component towards any notion of ‘Positive Ageing’. There have already been initiatives concerning increasing activity under the guise of ‘Active Ageing’ as part of the 2012 European Year of Active Ageing, which might combat sedentary working. Coupling this with wider considerations of social well-being through Positive Ageing initiatives might contribute also then towards the addressing of the UK societal difficulties associated above within the workplace. Thus, a need for fresh thinking about workplace research demands further attention towards Positive Ageing and which may represent a potential gap in current deliberations.

By undertaking an Structured Literature Review (SLR) as a scoping review, which might provide evidence of any potential argument for any sea-change towards more positive working practices, this paper considers the research question of what might be currently identified as ‘Positive Ageing’ and how is it represented. ‘Active Ageing’ might be separated historically as a concept, insofar as maintaining activity has been associated with positive outcomes for the ageing process and recognised in its own right. Within this paper, ‘Active Ageing’ is viewed as solely contributory to the overall field under consideration. Since this research seeks to think about the nature of the UK workplace rather than the health benefits of general activity relative to ageing, it is contemplated as part of ‘Positive Ageing’, rather than separately, for this scoping review.

The objectives of the study reported here were determined as: (1) to explore the nature of UK workplace ageing, and (2) to identify whether organisations incorporate the principles of ‘Positive Ageing’ as beneficial to both work productivity and resulting healthy ageing. By recognising ‘Active Ageing’ as contributory to ‘Positive Ageing’, to ascertain key relevant sources to the research questions and objectives presented and pursued, the study considered the relevance of current literature within the UK only. It was considered that this might countenance aspiration towards, or at a minimum, facilitate evidence towards any necessity towards re-thinking people management in the UK workplace by way of the needs of older workers. This might, in turn, allow a move towards positive approaches towards future workplace practices and identified ageing challenges.

## 2. Method

As an initial starting point, a SLR was progressed. Given constraints to the research to be undertaken, of time and of lack of funding, the following of a process incorporating research that seeks to identify prior sources from management/workplace writings (as opposed to occupational health sources) was identified as most appropriate to consider Positive Ageing within the practice of the UK workplace. By following the approach to the selection of key sources, the results of such review may then act as a model, to identify any gap between research and practice [8], which might inform further study and enquiry. This undertaking was enacted then by following the principles, as advocated by Tranfield et al. [8], who provide a scheme for the structure of a SLR by way of a methodology for developing evidence-informed management knowledge. The approach also considers the demands of producing an integrative literature review [9] as a distinctive form of research that uses existing literature to create new knowledge. By addressing the issue of the research–practice gap in human resourcing/workplace literature [4,7], it sought to be provocative to catalyse future research in terms of occupational health that might then provide by a marriage of the results of this initial enquiry, and to assess further directions for policy and practice.

As the review involved sources that are already within the public domain, no ethical clearance was required prior to this exploratory study being enacted. Using the search process, as determined by way of SLR [8], unless sources were identified as seminal, the research sought contemporary literature initially by the consideration of terms ‘current work practices’ and ‘older workers. PsychInfo, Psycharticles and Google Scholar for full text articles, published in the academic literature between the years 2008 and 2018, were searched using the search terms ‘Positive Ageing’ AND ‘workplace practices’. The time window to June 2018 provided the results as presented since it was recognised that there has been a major and unprecedented shift in economic conditions from the beginning of this period, which impacted the UK workplace (commonly referred to as the Global Financial Crisis (GFC)). Earlier research material may not take the impact of this shift upon the UK labour market or workplace practices into account, therefore, was discarded (with the proviso to include seminal work if it was identified). Rather than any review of ‘Active Ageing’ in the search criteria which was not the focus of the activity, following the guidance of Tranfield et al. (2003) in terms of following a Structured Literature Review approach [8], the search terms were extended to include ‘ageing’, ‘employment’, ‘unemployment’, ‘economic’, ‘productivity’ and all relevant combinations to attain the results presented. From this, key articles that reflected all the search terms and criteria were identified. In undertaking this review, it was recognised that there was extensive literature from Europe and Australia, which might be persuasive to the UK labour market practice. However, given that the scope of this study has UK challenges around their changed retirement age, non-UK literature was discarded, upon reading articles, it was determined that much of these can be found as informing the papers selected within their reference lists. They were discarded as holding limited relevance due to the differentiated nature of other labour markets, for example, lower ages of retirement in European countries or the comparative and representative nature of unskilled labour and skilled labour in Australia. It was also recognised as noted that, where there are key sources, for example, Institute for Employment Studies (IES), Department of Work and Pensions (DWP) or Chartered Institute of Personnel and Development (CIPD), these documents are informed by the wider studies but that these sources are incorporated into procedure or practice advocated in defence of chosen UK policies. The source documents of such non-UK papers identified were not incorporated, therefore, since it was determined that it is further the resulting policy and practice derived by the government agency, or other body, which is critical to the understanding of the enactment of Positive Ageing within the UK workplace.

Twenty different platforms were accessed (see Appendix B). Business Source Premier was accessed and the Equality and Human Rights Commission website, the Chartered Institute of Personnel and Development website. Publications were then prioritised for inclusion in the review in the following order:

- systematic reviews of previous publications and grey literature published outside of standard academic and/or commercial channels,
- empirical publications using data collected in the UK.

For empirical studies, the strength of the evidence was assessed. In line with and by following Brewis et al. [9], the following systematic review methodology was adopted as providing robust criteria within the SLR approach:

(i) the limitations identified by authors themselves,
(ii) author’s expertise in quantitative and qualitative methodologies—as quantitative or numerical data are usually collected through closed questions with fixed responses or produced by counting the number of times certain themes appear in qualitative data,
(iii) Qualitative data, expressed in words, are usually gathered using open questions with no fixed set of answers. This allows respondents to speak in their own words.
(iv) Any systematic reviews identified were evaluated by methodology and relevant coverage (level of depth of the examination) of the research topic, with the grey literature by methodology and policy relevance. For example, where a grey publication referred to empirical data but provided no details of the relevant study, this was identified as reducing its credibility. Practitioner-oriented (aimed at managers) given their broader applicability and suitability for audience were considered.

Related to the identified research questions, each source was summarised. In addition, it was recorded:

- whether the publication specified ‘Positive Ageing’, ‘Ageing’, or ‘Active Ageing’ (the focus was for Positive Ageing and Ageing–Active Ageing was not a focus of the study and therefore only identified for and to inform future reference/further research to be undertaken)
- whether it included details of method/s used, sampling and data analysis techniques for empirical work
- coverage and methodology for systematic reviews
- strengths and weaknesses identified by the author to determine whether a publication should be included in this report.

## 3. Results

In line with the systematic reasoning presented by Brewis et al. [9] and to clarify the limitations of the results presented, sources selected reviewed only evidence presented in English and excluded books, dissertations and theses solely due to their length given the limited time available. Search results identified 58 papers concerning ageing, based upon the overall criteria for this report (see Table 1)—of which 40% were considered as potentially determined as empirical studies in terms of ageing, for example, including statistical analysis of demographic shifts. Of the empirical studies, only 10% are based on primary data; 40% use quantitative data; and 90% were based on data from the UK (see Table 2). From this, the SLR was applied to 35 sources (see Appendix A), selected upon a further reading based upon thematic selection from the terms of the search. The further selection process was based upon the identified sources as representative of ‘employment’, with the final categorisation incorporated as those reflecting ageing through ‘economic’, ‘productivity’ terms, for example, business case. The articles were also selected where it was recognised that there were other sources informing these policy statements, or recommendations, derived from the identified key sources from the initial 58 articles. The incorporation of any additional wider selection would not have provided greater benefit to the outcomes of the literature review for the audience. Given the interrelated themes within the final selection and those excluded, the audience might still be able to access sources (by the reference lists of the final articles) if necessary to their own research. It is recognised that the interpretation of sources appropriate to the results presented relies upon the SLR process followed but that the rigour of the outcomes is reliable.

Full details of all selected publications appear in the reference list then and Appendix A provides details of the SLR results for this scoping review. Not all the reviewed evidence defined the terms it uses. Furthermore, the evidence reviewed does not use consistent terminology. There is some interplay in definitions and terms. Whilst the term ‘active’ might be viewed firstly as referring to physical activity, it was determined then that this also refers to positive levels of activity. There was a relationship between themes and, at times, contradiction between terms surrounding ‘Positive Ageing’. By examining results to determine the contributions that closely matched the issues of work practices and ageing, rather than any wider application in terms of activity was pursued also to facilitate further refinement to provide the final discussion. Several strands of discourse were reviewed from the sources identified. These are presented in the following discussion therefore. However, in recognition of the limitations of the process as described above, it is also declared that in terms of research parsimony, the defence for the methodological approach applied lies in line with SLR [8] and reasoning of Brewis et al. [9].

## 4. Discussion

From the scrutiny of literature, it was apparent that, within the UK, the labour market participation rate for people aged between 50 and 65, has reached 75.3% [10]. Positive Ageing may be accepted as resulting in increased economic capacity. This potentially saves costs in terms of wider public healthcare providers as a direct consequence of employing older workers. Yet the UK government, as part of its future industrial strategy, recognises that there remains challenges for society, and the labour market, in terms of this shifting demographic and seeks to engage with such challenges [11].

Remaining work active and contributing economically further offer the potential to reverse demoralising ideas that older people are a burden on society. There are possible views that, whilst it might be levelled that the needs of business might lie with the financial ‘bottom line’, business itself is not separate from society. As society shifts so business practice needs to as well. Extending such notions might potentially allow for a review of work practices in terms of later life, and potentially might reveal unanticipated benefits to productivity by engaging with older employees in ways that maximise their personal benefit from and potential contributions. Historically, it might be observed then that such relationship between economic contribution and positive views around ageing workers may have lacked prior scrutiny. Thinking has been limited solely to the scope of capitalistic ‘financialisation’ of the firm outside society and foresaw age as a burden.

The inclusion of ‘Positive Ageing’ repositions the individual worker, both within their potential for reducing costs to society, alongside increased potential productivity and profitability for business. This potentially opposes any current motivation within people and workforce management as lying solely with the aspiration of increasing profit and as blind to the contribution of the ageing worker. This is especially poignant where historical workplace motivations have proven to be at the detriment of the older workforce. The adoption of ‘Positive Ageing’ as a people management approach or a strategy might reverse such impediment and, by contrast, increase the perceived economic value of the ageing individual. The adoption of principles of ‘Positive Ageing’, (with ‘Active Ageing’ to combat workplace inactivity) can also help to refocus societal thinking about ageing. Not only as it relates to the workforce but also as it addresses comprehensive measures which might enable and encourage people to continue working for longer. This would then support further the potential sustainability of pension systems [9]. Significantly Positive Ageing shifts strategic planning from a needs-based approach to a rights-based approach. This recognises the rights of people to equality of opportunity and treatment in all aspects of their life [10].

Women in the UK have retired earlier historically than men. They are then especially more likely to be affected by a changed national default retirement age. Women also traditionally carry the burden of caring responsibilities. Thus, the prospects of ageing may be very daunting. There remains a dearth of writing regarding women who have ended up in low qualification and low paid jobs and who struggle financially. Despite that, working women from this category are more likely to leave work if they receive lower pay compared to average wage levels, feel isolated at work or are the victim of sexism in the workplace. Such workers may also face long-term unemployment and lack training opportunities [12,13].

Concepts of Positive Ageing are applicable towards any consideration of maintaining health and functional capacity in work. The adoption by workers of a variety of healthier habits can promote improved lifestyles. Due to changes in health, and functional capacities induced by ageing, there still needs to be repeated articulation of the need for a fundamental understanding about the adjustments needed at work [14].

Using social identity theory, those who cognitively identify themselves as ageing, benefit, i.e., acknowledge their limitations (for example, physical decline), as compared alongside any advantages (for example, increased expertise). A correlation between a person’s positive cognitive and affective identification of themselves as an ageing worker and successful ageing in the workplace can be established [15]. Older workers have greater commitment than their younger colleagues, and a positive emotional relationship between age and work engagement exists [16]. The impact of negative age-related stereotypes and social norms around the workplace by contrast, however, recognises that older workers are discouraged by negative stereotype threats. They sought early retirement as their aspirations were diminished by views about their abilities to work, learn and develop—this was most likely to be found in the West, as opposed to more virtuous Eastern societal views towards older workers [17]. A possible current fashion of the UK, which might be attributed to ageism, perhaps, a ‘cult of youth’ in employment, means some Talent Management strategies largely focus upon establishing ‘who is talent’ from early engagement in the organisation, for example, graduate training. By contrast, viewing talent from this life span perspective [16], older workers may show that strong obligation represents a positive relationship between age and work engagement. This essentially reflects that older and more experienced workers are likely to be more dedicated to their career—which may, or may not be, dependent upon any single employer, nor upon the acts of one employer. This is not widely recognised culturally in the UK.

In terms of career advancement through talent management strategies, most menopausal women have been identified as being placed on the “sticky floor”. This reflects limitations to their career and has often led to them giving up full time employment before 67 [18]. Despite more fashionable rhetoric of expectations of frictional employment, by way of career models which assume that all employees will seek to participate in an ‘up-or-out tournament’ in a gig economy; in reality, employment in the UK currently reflects an average period of employment of 10 years per employer or role [19,20,21]. In part, due to labour market supply or demand, this might be attributed to barriers to exit within employment. Another interpretation might recognize the positive relationship which is being depicted by employees gaining job resources with age. This provides them with coping mechanisms, as well as their skills in mediating their tenure even during any period of difficulty in the workplace—for example, austerity in post global financial crisis, or BREXIT climate. Despite age discrimination being made illegal through introduction of the Equality Act 2010, it remains largely a modern, post WWII phenomena within the UK. Prior to that, older workers were historically depicted as both wise and loyal [21].

As previously noted, however, UK employers do not make the most of the assets of their ageing workers, let alone actively promote ‘Positive Ageing’. However, enhanced knowledge acquired by older workers through experience may compensate dwindling functional abilities. Ageing can be an opportunity for mental growth, as opposed to decline, several factors can improve with age. For example, strategic thinking, sharp-wittedness, considerateness, wisdom, ability to deliberate, ability to rationalise, control of life, holistic perception and language skills can all be determined as success stories for the ageing process. Older workers are also committed and engaged with their work. They are loyal towards their employer. They often record less absenteeism than other age groups. Work experience can compensate for the decline of some basic cognitive processes, such as memory functions and psychomotor skills and improve the valuable social capital of older workers. Additionally, professional competence, tacit knowledge, cooperation skills grow, structural awareness about the organisation all improve with longer working. This is alongside extending customer contacts and networks that expand over time, together with understanding about changes in operational environment improving [22].

Important elements towards adoption of the ‘Positive Ageing’ approach described can also help to overcome prejudices towards ageing workers. Older workers have a right to good health, to lifelong learning and to participate in economic growth. Thus, strategies to promote positive experiences in work will strengthen people’s attitudes and values towards the job. This includes promoting trust in the person’s employer, support and feedback of supervisors, fair treatment, and engagement with work [22]. As values, attitudes and motivation are not often the target of direct interventions but are more likely to be influenced more indirectly. People should feel that they are respected, that they can trust their employer. This becomes a responsibility of workers themselves, in terms of their values, attitudes and other personal factors [22]. Rather than focussing upon the peculiar needs of older workers, this thinking might be argued as just good management strategies for all.

A contrasting and more desirable approach for a proactive line is apparent in organisations who are prepared to enhance individual resources and to support intergenerational learning. This equates to a life-course approach that might create equal opportunities for all generations [22]. Proactive approaches may be influential for older workers’ perceptions of ability/inability to continue in work.

There has been also continued concern about increasing work intensification in the UK [23]. Despite UK legislation making age a protected characteristic, negative workplace management cultures persist and are age unfriendly [24]. Strategies towards people management have led to the abandonment of best practice UK workplace processes in the 21st century. Such negative cultures may lead to adverse lifestyle behaviours being forged in workplaces, such as sedentary lifestyles. Work intensification can result in the manifestation of raised stress levels. Moreover, there are considerable concerns about mental health problems evolving from the UK workplace (burnout/exhaustion) then transferring into the period of retirement and the lack of reasonable adjustments which might be enacted for an ageing worker [25,26].

The negative management of ageing in the workplace is compounded by problems of bigotry and discrimination associated with ageism [27]. Age is a fluid social construct characterised by transition through different stages of the lifespan. Some of the issues faced when considering older people and their related work lie with this age discrimination. For the purposes of re-shaping thinking and cultural bias towards a younger labour force, such discrimination should be named and challenged.

The boundary conditions of the current fluid workplace moreover may be illustrated by the fuzzy nature of UK personal life and work life. For example, the personal use of technology impacts upon the job demands and resources by different workers [28]. This may have both positive and negative organisational outcomes. This might not be determined by ageing but through tensions between flexibility and control for the workforce. In terms of technological usage, perhaps age discrimination predominates in organisational discourse by the viewing of work by those in later life, however, in its usage. However, this may disguise the reality of such tensions. Such acts of bigotry merely reflect ‘prejudicial’ viewings of the nature of older workers, for example, through suggestions that they are unable to engage with technology, when the technological issues might be universal in terms of the limitations of the employment of the same technology by others. This might not have been uncovered at first. This remains raw discrimination however.

The importance of wisdom has been acknowledged throughout history leading to soundness of judgement. However, despite the rise of the importance of wisdom across cultures, the relevance of significance of the ‘wise person’ (read, older person) has become less revered in the UK [29]. To some degree, the nature of an individual is based, however, in their personal evaluation of their own skills or experience. If the employee feels that their competence exceeds role demands of the work they are employed within, then this can trigger feelings of relative deprivation. Withdrawal symptoms are exhibited when the person feels that they exceed the qualifications or skill set required. This may lead to a sense of age discrimination as compared to those who feel less qualified for their role. Perceptions of age discrimination can depend upon the threshold of the individual by way of their personal toleration of treatment, which they might still consider as negative [30]. Despite limited empirical examples, some commitments are provided from major employers such as McDonalds, Wetherspoons or Lloyds Bank by way of their policies towards workplace practice [31] that are presented as an attempt to challenge notions of age discrimination.

It is evident that an increase in physical and mental health problems (e.g., musculoskeletal problems and mental problems such as depression) as a feature of ageing should not be ignored in this debate however. These require reasonably achievable adjustments at work to prevent the risks of work inability and early retirement [32]. They should not be viewed by employers as burdensome or requiring additional costly resources. It may be possible to markedly postpone the development of ageing changes in workers’ bodies without major investments. Positive Ageing has multiple strands. It incorporates genuine practical adjustments for the conditions of work but also that there is an overarching need to consider the underpinning nature of bigotry and discrimination found in the UK workplace—without which, the benefits and value older workers offer may be valued appropriately. Despite much of the discussion of inclusion and diversity providing a broad brush in terms of all the protected characteristics of employees further to the Equality Act of 2010, the value of older workers need not be undermined due to discrimination that needs addressing [33]—to which this review would benefit from extension in terms of occupational health literature, by the development of the fuller examination from the health perspective, and by way of the marriage of these issues illustrated from the policies and issues challenging the workplace in terms of re-thinking later life working or the considerations of older workers.

## 5. Conclusions

This review has drawn from a structured literature review (SLR) conducted and which provides evidence-based knowledge to highlight the need for greater awareness of wider ‘Positive Ageing’ related issues in the workplace. The paper presented should be recognised as limited by the constraints of time and funding, without which potentially a more exhaustive review might have been attained to assure reproducibility. The review was completed by the first half of 2018. Any later literature published will not be contained. It might still be concluded that there is a need to increase understanding towards actuating ‘positive’ ageing for both the benefit of individuals, with less attention to concerns for profit-centric commercial economic stability but recognising the business case for so doing. Culturally, currently in the UK, there is little consideration of the long- term management of people by the way of the adaption of their work-life outside central government policy initiatives. For the needs of business and society to engage in a mutually beneficial stance this needs to be progressed, a dearth of current empirical research was identified at the undertaking of this review. To move away from the possible position of age bigotry, people management practices need thinking to move from policy makers into practice. The sources identified by this study provide opportunities to commence some discourse which might think more widely about the issues, to stimulate recognition for the need to undertake further empirical research and to review adaptations in work-life also by recognising the evidence of a gap in thinking about older workers. It further recognises the challenges for society if this gap prevails not only in the business case presented by way of a national industrial strategy but further in terms of positive and healthy ageing as lifelong sedentary work and work intensification represents future health problems for our youth, rather than a matter solely of later life and economic inactivity of older workers.

## Figures and Tables

**Table 1:** Criteria for article selection.

Criteria	Explanation
Current research	UK sources
Where published	Government publications Business Journals Age Related Organisations
Who published it	Academic papers or relevant and legitimate business organisations
Relevance of Article	Articles which fell within the scope of the study
Time Frame	2009 to 2018
Number of Articles	30 identified

**Table 2:** Details of the evidence base criteria: percentages reflected were rounded up.

Data Type	Empirical Studies	Non-Empirical Grey Literature Includes Government Reports, Policy Statements and Issues Papers, Conference Proceedings	Systematic Reviews	Systematic Reviews and Empirical Studies
**Percentage**				
**Primary data**	10%	5%	0%	10%
**Secondary data**	30%	50%	100%	80%
**Both**	40%	55%	100%	100%
**Quantitative**	40%	50%	40%	80%
**Qualitative**	20%	25%	10%	30%
**Both**	10%	10%	5%	10%
**Cross-sectional**	50%	25%	25%	50%
**Longitudinal**	10%	5%	5%	5%
**Mixed design**	20%	10%	10%	10%
From the UK *	90%	90%	90%	90%

* The review incorporated ONS (Office for National Statistics) which refers to the UK labour market statistics and included as pertinent within the constraints of the scope of this study. The European Commission Source [1] represented a source outside the UK but was included for the same reasoning.

## Appendix A. List of Papers (Thematic) Final Analysis of Sources

**Table:**

Articles focussed upon Positive Ageing Incorporating Active Ageing and Ageing	1. European Commission. *The 2018 Ageing Report Underlying Assumptions and Projection Methodologies*; European Commission: Institutional Papers, November 2017. Available online: https://ec.europa.eu/info/sites/info/files/economy-finance/ip065_en.pdf (accessed on 1 December 2017) *. 2. *Future of an Ageing Population*; Government Office for Science: London, UK, 2016. Available online: www.gov.uk/go-science (accessed on 21 December 2017). 3. UK Labour Market, December 2015. Available online: https://www.ons.gov.uk/employmentandlabourmarket/peopleinwork/employmentandemployeetypes/bulletins/uklabourmarket/december2015 (accessed on 21 December 2017) *. 4. HM Government. *Industrial Strategy*: *Building a Britain Fit for the Future*; White Paper, HMS, 2017. Available online: https://www.gov.uk/government/publications/industrial-strategy-building-a-britain-fit-for-the-future (accessed on 1 December 2017). 5. Centre for Ageing Better. *Later Life in 2015: Analysis of Views and Experiences of People Aged 50 and Over*; Ipsos MORI, 2017. Available online: http://laterlife.ageing-better.org.uk/resources/cfab_lli_2015_ipsos_mori_report.pdf (accessed on 1 December 2017). 6. Warr, P. Work values: some demographic and cultural correlates. *J. Occup. Organ. Psych.* **2008**, *81*, 751–775. 7. Drydakis, N. *Measuring Age Discrimination in the UK: A Field Experiment for the 2013–2015 Period*; A technical report produced for the Chartered Institute of Personnel and Development. Anglia Ruskin University: Cambridge, UK, 2015; pp. 1–4. 8. Sargent-Cox, K. Ageism: we are our own worst enemy. *Int. Psychogeriatr**.* 2017, *29*, 1–8. 9. Mitchell, L.K.; Knight, B.G.; Pachana, N.A. Wisdom across the ages and its modern day relevance. *Int. Psychogeriatr.* **2017**, *29*, 1231–1234. 10. Wuthrich, V.M. Changing our thinking about changing their thinking in older adulthood. *Int. Psychogeriatr.* 2017, *29*, 1405–1407. 11. Delhom, I.; Gutierrez, M.; Lucas-Molina, B.; Meléndez, J.C. Emotional intelligence in older adults: Psychometric properties of the TMMS-24 and relationship with psychological well-being and life satisfaction. *Int. Psychogeriatr.* **2017**, *29*, 1327–1334. 12. Wainwright, T.; Kibler, E. Beyond financialization: Older entrepreneurship and retirement planning. *J. Econ. Geogr.* **2014**, *14*, 849–864. 13. Kautonen, T.; Hatak, I.; Kibler, E.; Wainwright, T. Emergence of entrepreneurial behaviour: The role of age-based self-image. *J. Econ. Psychol.* **2015**, *50*, 41–51. 14. Ainsworth, S. Aging Entrepreneurs and Volunteers: Transition in Late Career. In *Aging Workers and the Employee-Employer Relationship*; Bal, P., Kooij, D., Rousseau, D., Eds.; Springer, Cham, Switzerland, 2015. 15. Funken, R.; Gielnik, M.M. Entrepreneurship and Aging. In *Encyclopedia of Geropsychology*; Pachana, N., Ed.; Springer: Singapore, 2015; pp. 1–7.
Articles identified as ‘employment’, and ‘unemployment’ relative to ageing	16. BITC–Business in the Community. *The Missing Million: Illuminating the Employment Challenges of the over 50s*; Research Report by the International Longevity Centre UK (ILC-UK); The Prince’s Responsible Business Network, 2014. Available online: https://age.bitc.org.uk/all-resources/research-articles/missing-million-report-1 (accessed on 1 December 2017). 17. Tkachenko, O.; Hahn, H.-J.; Peterson, S.L. Research–Practice Gap in Applied Fields: An Integrative Literature Review. *Hum. Resour. Dev. Rev**.* **2017**, *16*, 235–262. 18. Kristjuhan U. Ergonomics as a tool in prolonging youth and postponing ageing. *Work* **2012**, *41*, 380–382. 19. Loretto, W.; Vickerstaff, S. The domestic and gendered context for retirement. *Hum. Relat.* **2013**, *66*, 65–86. 20. Loretto, W.; Vickerstaff, S. Gender, age and flexible working in later life. *Work, Employment and Society* **2015**, *29*, 233–249. 21. Taylor, P.; Loretto, W.; Marshall, V.; Earl, C.; Phillipson, C. The older worker: Identifying a critical research agenda. *Social Policy and Society* **2016**, *15*, 675–689, doi:10.1017/S1474746416000221.
Articles addressing ageing through ‘economic’, ‘productivity’	22. Centre for Ageing Better. *Response to Work and Health Green Paper,* March 2017. Available online: http://www.ageing-better.org.uk (accessed on 1 December 2017). 23. Rudawska, I. Active Ageing and its Impact on Labour Market. *Economics & Sociology* **2010**, *3*, 9–24. 24. Cheung, F.; Wu, A.M.S. Social Identification, Perception of Aging, and Successful Aging in the Workplace. *Journal of Career Development* **2014**, *41*, 218–236. 25. Kim, N.; Kang, S.-W. Older and More Engaged: The Mediating Role of Age-Linked Resources on Work Engagement. *Hum. Resour. Manag.* **2017**, *56*, 731–746. 26. Morgan, M.S. *Glass Ceilings and* *Sticky Floors**: Drawing New Ontologies*; Economic History Working Papers No: 228/2015; School of Economics and Political Science, Economic History Department: London, UK, December 2015. 27. Malhotra, N.; Morris, T.; Smets, M. New career models in UK professional service firms: from up-or-out to up-and-going nowhere? *Int. J. Hum.* *Resour. Manag.* **2010**, *21*, 1396–1413. 28. Gallian, M.; Desmette, D. (In)Validating Stereotypes about Older Workers Influences their Intentions to Retire Early and Learn and Develop. *Basic and Applied Psychology* **2010**, *32*, 86–98. 29. McBride, A. Lifting the Barriers? Workplace Education and Training, Women and Job Progression. *Gender, Work and Organization* **2011**, *18*, 529–547. 30. Rittenhofer, I.; Gatrell, C. Gender Mainstreaming and Employment in the European Union: A Review and Analysis of Theoretical and Policy Literatures. *Int. J. Manag. Rev.* **2012**, *14*, 201–216. 31. McCann, L.; Hassard J.; Morris, J. Restructuring Managerial Labour in the USA, the UK and Japan: Challenging the Salience of ‘Varieties of Capitalism’. *British Journal of Industrial Relations* **2010**, *48*, 347–374. 32. CIPD. Creating and capturing value at work: who benefits? Part 1. Available online: www.cipd.co.uk (accessed on 21 December 2017). 33. Nicholson, P.J.; Mayho, G.; Robson, S.A.; Sharp, C. *Ageing and the Workplace:* *A Report from the BMA Occupational Medicine Committee 21* *September 2016*; British Medical Association (BMA): London, UK, 2016, pp. 1–32 *. 34. Sungdoo, K.; Christensen, A.L. The Dark and Bright Sides of Personal Use of Technology at Work: A Job Demands–Resources Model. *Hum. Resour. Dev. Rev**.* **2017**, *16*, 425–447. 35. Triana, M.C.; Trzebiatowski, T.; Byun, S.-Y. Lowering the Threshold for Feeling Mistreated: Perceived Overqualification Moderates the Effects of Perceived Age Discrimination on Job Withdrawal and Somatic Symptoms. *Resour. Manag.* **2017**, *56*, 979–994. 36. Institute of Employment Studies (ies). *What do Older Workers Value about Work and Why?* Centre for Better Ageing, 2017; pp. 1–26. Available at https://www.ageing-better.org.uk/sites/default/files/2017-12/What-do-older-workers-value.pdf (accessed on 21 December 2017). 37. *Fuller Working Lives: A Framework for Action*; Department for Work and Pensions (DWP), 2014. Available online: https://www.gov.uk/government/publications/fuller-working-lives-a-framework-for-action (accessed on 1 December 2017). 38. CIPD. *Diversity and Inclusion at Work: Facing up to the Business Case*; CIPD report June 2018. Available online: https://www.cipd.co.uk/Images/diversity-and-inclusion-at-work_2018-summary_tcm18-44150.pdf (accessed on 1 July 2018). 39. Thomas, A.; Pascall-Calitz, J. *Default Retirement Age – Employer Qualitative Research*; Department for Work and Pensions, Research Report No 672, 2010. Available online: https://www.gov.uk/government/publications/default-retirement-age-employer-qualitative-research-rr672 (accessed 1 December 2017).

* The European Commission included for reference to UK statistical profile and not considered as a European or study from other overseas source. ** the inclusion of article from BMA (British Medical Association) was not considered as an ‘occupational health’ source as it contains evidence around prejudicial working for older workers and attitudes.

## Appendix B

Grey literature platforms *

Positive ageing in the Workplace

1. World Health Organisation. *World Report on Ageing and Health* *2015*. Available online: http://www.who.int/ageing/events/world-report-2015-launch/en/ (accessed on 1 December 2017).
2. The Chartered Institute of Personnel and Development. Available online: www.cipd.co.uk (accessed on 21 December 2017).
3. Department for Work and Pensions. Available online: https://www.gov.uk/government/organisations/department-for-work-pensions (accessed on 23 December 2017).
4. Institute for Employment Studies. Available online: https://www.employment-studies.co.uk/resource/fulfilling-work-what-do-older-workers-value-about-work-and-why (accessed on 1 December 2017).
5. British Medical Association (BMA). Available online: www.bma.org.uk (accessed on 1 December 2017).
6. Equality and Human Rights Commission. Available online: https://www.equalityhumanrights.com/en/search?text=ageing (accessed on 21 December 2017).
7. Centre for Ageing Better. Available online: http://www.ageing-better.org.uk (accessed on 21 December 2017).
8. International Longevity Centre UK (ILC-UK), The Prince’s Responsible Business Network, BITC–Business in the Community. Available online: https://www.bitc.org.uk/ (accessed on 23 December 2017).
9. National Institute of Health and Care Excellence. Mental wellbeing in over 65s: occupational therapy and physical activity interventions. Available online: https://www.nice.org.uk/guidance/ph16/resources (accessed on 23 December 2017). Note: Not used in final review as not related to workplace.
10. HM Government, Industrial Strategy: Building a Britain fit for the future, White Paper, HMS, 2017. Available online: https://www.gov.uk/government/publications/industrial-strategy-building-a-britain-fit-for-the-future (accessed on 1 December 2017).
11. Government Office for Science. Available online: www.gov.uk/go-science (accessed on 1 December 2017).
12. British Psychological Society, Age and Ageing. Available online: https://www.bps.org.uk/topics/age-and-ageing (accessed on 1 December 2017).
13. Ageing and life course. Available online: http://www.who.int/ageing/healthy-ageing/en/ (accessed on 1 December 2017).
14. Sport England, Positive Ageing Programme. Available online: https://www.hse.ie/eng/about/who/healthwellbeing/our-priority-programmes/positive-ageing/ (accessed on 1 December 2017).
15. European Commission. The 2018 Ageing Report: Underlying Assumptions & Projection, Methodologies, Institutional Paper No 65, November 2017. Available online: https://ec.europa.eu/info/sites/info/files/economy-finance/ip065_en.pdf (accessed on 23 December 2017).
16. Department of Health, Healthy Ireland. National Positivie Ageing Strategy. Available online: https://health.gov.ie/healthy-ireland/national-positive-ageing-strategy (accessed on 1 December 2017).
17. The Health Service Executive, Ireland. Positive Ageing Starts Now! The National Positive Ageing Strategy. Available online: https://health.gov.ie/wp-content/uploads/2014/03/National_Positive_Ageing_Strategy_English.pdf (accessed on 23 December 2017).
18. Better Health Channel, Healthy and Active Ageing Victoria State Government. Secrets to healthy ageing. Available online: https://www.betterhealth.vic.gov.au/health/ten-tips/Healthy-ageing-tips (accessed on 21 December 2017).
19. Positive Ageing in London. The London Regional Forum for Older Londoners (50+). Available online: http://pailondon.org.uk/who-we-are/ (accessed on 1 December 2017).
20. BBC, BBC Radio 4, You and Yours, When I’m 65–Positive Ageing. Available online https://www.bbc.co.uk/programmes/p00vlddv (accessed on 21 December 2017).

* These platforms were sought however those material outside of the UK were reviewed in terms of relevance to the UK only and not necessarily included in the final literature review (as per methodology stated). There was a limitation at 20 since there are extensive sites worldwide, but the literature review focused upon the UK. Sites outside UK listed above were reviewed as grey literature solely to inform the final selection process within the SLR.
